# Influence of Infill Density on the Fatigue Performance of FDM-Manufactured Orthopaedic Plates

**DOI:** 10.3390/ma19040816

**Published:** 2026-02-20

**Authors:** Aleksa Milovanović, Simon Sedmak, Aleksandar Sedmak, Filip Vučetić, Katarina Monkova

**Affiliations:** 1 Innovation Centre of the Faculty of Mechanical Engineering, Kraljice Marije 16 Street, 11120 Belgrade, Serbia; ssedmak@mas.bg.ac.rs (S.S.); fvucetic@mas.bg.ac.rs (F.V.); 2 Faculty of Mechanical Engineering, University of Belgrade, Kraljice Marije 16 Street, 11120 Belgrade, Serbia; asedmak@mas.bg.ac.rs; 3 Faculty of Manufacturing Technologies with a Seat in Presov, Technical University of Kosice, Sturova 31, 080 01 Presov, Slovakia; 4 Faculty of Technology, Tomas Bata University in Zlin, Vavreckova 5669, 760 01 Zlin, Czech Republic

**Keywords:** orthopaedic plates, additive manufacturing, FDM, infill density, PLA material, numerical simulation

## Abstract

Orthopaedic plates are long-established medical devices conventionally manufactured from metals, most notably titanium alloys. The introduction of Additive Manufacturing (AM) has created new opportunities to design implants with complex internal architectures, enabling precise control over infill patterns and densities that directly influence mechanical properties and fatigue performance. Biodegradable polymers such as polylactic acid (PLA) have attracted growing interest in biomedical engineering, potentially reducing the need for secondary implant-removal surgery if degradation rates are carefully controlled and clinically approved. Additionally, AM offers the ability to customise internal structure for improved mechanical performance and load-bearing, while also providing the possibility of integrating advanced functionalities, such as controlled drug delivery. Building on previous work by our research group at the University of Belgrade, this study investigates the fatigue behaviour of the best-performing AM-optimised orthopaedic plate design. Numerical models incorporating honeycomb infill structures with the full range of achievable densities were developed to assess structural integrity under fatigue loading. Fatigue crack growth was simulated in ANSYS Mechanical (ANSYS Inc., Canonsburg, PA, USA) software, employing a four-point bending configuration in accordance with the ASTM F382 standard. A validated PLA material model was implemented at a reduced load level (10%) relative to previous studies. Direct comparison with titanium plates was avoided due to fundamentally different material properties, focusing instead on infill architecture to identify optimal AM design strategies for orthopaedic plates.

## 1. Introduction

Polylactic acid (PLA) material is the most extensively studied biodegradable polymer to date, widely regarded in industry as a leading candidate for replacing conventional petrochemical-based polymers. Another important role of PLA is its implementation in Additive Manufacturing (AM), due to its ease of processing. Owing to its relatively high strength and stiffness among polymers, PLA is not only suitable for prototyping but also for certain functional applications, and it is currently under investigation for a broad spectrum of medical uses [1,2]. PLA was first approved by the Food and Drug Administration (FDA) for packaging applications, along with several other polymers [3]. Since then, it has been investigated for a wide range of biomedical applications, including barrier membranes [4], drug delivery systems [5,6,7], bone scaffolds [8,9,10], tissue engineering [11,12], orthopaedic applications [13,14,15], and absorbable staples, sutures, screws and pins for bone fixation [3,16].

In addition to applications in regenerative medicine, cardiovascular, orthopaedic, and dental fields, PLA is also considered for the fabrication of medical equipment. This polymer is biocompatible, potentially recyclable under controlled conditions, and compostable with no evidence of carcinogenicity in approved biomedical applications. Its fabrication requires significantly less energy than petroleum-based polymers, and it degrades primarily into lactic acid, a generally biocompatible product whose local biological effects depend on the degradation rate and conditions [17,18]. Furthermore, the material may be suitable for drug-delivery applications, as controlled degradation can be achieved through tailored material composition and processing conditions. This feature provides for sustained, long-term release of therapeutic agents in scaffolds. Additionally, PLA-based drug-delivery systems have demonstrated considerable potential in various medical treatments, including innovative approaches in cancer therapy [5,17,19]. The potential for load-bearing applications is also a benefit of PLA; however, its biodegradable nature makes it particularly attractive for short-term orthopaedic plates, where gradual degradation following bone healing is advantageous [3].

Given these potential applications, PLA’s mechanical properties and fracture behaviour have been extensively studied in recent years. Due to PLA’s popularity in Fused Deposition Modelling (FDM), most studies have focused on the influence of printing parameters on final component properties. Table 1 in ref. [1] provides general guidelines for the mechanical properties of injection-mould-grade PLA, with the following representative properties: tensile strength of 59 MPa, elongation at break of 7%, and elastic modulus of 3.5 GPa. The thermal properties of PLA, reported in [19], include a glass transition temperature of 54–59 °C and a melting temperature range of 170–178 °C.

Most FDM processes cannot achieve the mechanical properties of injection-moulded PLA; however, some studies report that up to 85–90% of the tensile strength can be attained at high nozzle temperatures of 250 °C [20,21]. Since FDM is employed in both hobbyist and industrial machines, such temperatures are excessive for most low-cost systems and may lead to filament clogging. A more practical nozzle temperature range of 170–210 °C has been discussed in [22], with optimal properties observed at 190–210 °C for low printing speeds (e.g., 40 mm/s). Geometrical accuracy is generally improved at the lower end of this range. Layer height also plays a significant role, with 0.1 mm layers providing the best overall properties, albeit at the expense of longer production times [21,23]. Mechanical properties are highly anisotropic, emphasising the importance of raster orientation; for tensile testing, rasters aligned with the load direction yield superior results [21,23,24,25,26]. Finally, the infill pattern and density are critical parameters for tailoring the internal architecture of FDM components and have a substantial impact on their mechanical properties [27].

Due to the brittle nature of PLA [3], most fracture assessment studies have relied on Linear Elastic Fracture Mechanics (LEFM) rather than the Elastic–Plastic approaches. The influence of process parameters is also evident in fracture tests, which are commonly conducted according to ASTM D5045 [28] and ASTM D6068 [29] using Single Edge Notched Bending (SENB) and Compact Tension (CT) specimens. A comprehensive study by [30] examined raster and build orientations, layer height, filament colour, and machine type, demonstrating that all investigated process parameters significantly affect the fracture properties of PLA material. Similarly, ref. [31] investigated the effects of build orientation and process speed on CT specimens, showing that lower speeds lead to higher fracture toughness values. Regarding raster orientation, ref. [32] reported an inverse correlation between tensile properties and fracture toughness. In particular, the −45°/45° orientation showed an approximately 30% increase in fracture toughness compared to the 0° orientation, which is aligned with the loading direction.

Beyond process parameters, specimen preparation can also influence fracture assessment results. Namely, research led by [33] reported that directly printing the notch, rather than milling it, reduces data scatter, highlighting the importance of notch fabrication methods in obtaining reliable fracture property measurements. Additionally, crack straightness during testing must be ensured, with perimeter lines shorter than the pre-crack, thereby preventing deviation from the intended crack path [34,35]. The effect of infill pattern and density remains insufficiently explored, with only a limited number of studies addressing their influence on fracture properties [36,37].

The most important aspect for functional applications of AM materials is the assessment of fatigue properties. A thorough study by [38] reported that infill density had the greatest influence on component lifespan, followed by layer height, nozzle diameter, and process speed, in that order. The study employed a Taguchi orthogonal array to identify the relative significance of these parameters. Similarly, ref. [39] showed that process speed is the least influential factor, indicating that faster manufacturing speeds can be achieved without significantly affecting the resulting properties. Both studies [38,39] used a honeycomb structure as the internal architecture. In contrast, ref. [40] observed a notable influence of process speed, which was likely a consequence of the large disparity between the investigated speeds (20 and 80 mm/s). Since FDM components inherently contain a high concentration of voids, lower layer heights produce a greater contact area between layers with smaller voids, thereby improving structural integrity [41,42]. Furthermore, ref. [43] reported that lower layer heights also promote straighter crack paths, as observed on CT specimens.

Polymers may experience fatigue failure through either thermal or mechanical mechanisms. Thermal failure occurs when hysteretic heating causes local softening or melting of the material, whereas mechanical failure is driven by the initiation and propagation of the crack under cyclic loading. For this reason, fatigue tests are typically performed at frequencies between 1 and 25 Hz, with recommendations to remain below 5 Hz to minimise heat generation [42]. Nevertheless, several studies confidently used 10 Hz in their experiments without reporting any thermal issues [43,44,45]. The fracture mechanical aspects of fatigue in PLA were examined by [46], who reported that exceptionally good fusion between layers and strands produced a nearly homogeneous structure, resulting in similar fatigue performance across all raster orientations. Both crack initiation and propagation laws were considered, with crack-growth kinetics described using Paris’ law [47]. These findings were subsequently used to estimate the lifetime of a PLA structural component, combining Finite Element Analysis (FEA) with experimental methods to model such complex structures [48].

In certain functional applications of AM, particularly biomedical implants, the internal architecture must be carefully tailored to allow tissue growth and osteoinduction [39]. In these cases, a compromise has to be found between mechanical performance and biological functionality. A key advantage of FDM in this context is, first of all, the use of biocompatible and biodegradable materials for component manufacturing, and, second, the ability to precisely manipulate infill patterns and densities. This approach also allows the production of drug-delivery systems with controlled drug-release kinetics, which is especially interesting for new orthopaedic plate designs [5,17,19].

To date, Finite Element Analysis (FEA) has been widely used to predict the lifetime of conventional metallic implants. Numerous well-established designs, including hip, knee, dental implants and orthopaedic plates, have been extensively evaluated using this method [49,50,51,52,53,54,55,56,57,58,59,60,61]. In recent years, the research focus has shifted toward assessing AM components for such purposes, despite their tendency to contain inherent defects [62,63,64,65]. These properties introduce unique challenges for accurate lifetime prediction in biomedical applications. Moreover, polymeric materials represent a promising alternative due to the advantages mentioned above, although their viscoplastic, time- and rate-dependent behaviour must be carefully considered. In line with previous research on titanium alloy orthopaedic plates [66,67,68], this study adopts PLA as the material of choice for orthopaedic plate design. Based on the conducted FEA, among the five available plate geometries, the design exhibiting the greatest fatigue life, corresponding to the highest bone–plate contact, was selected. This plate geometry was combined with a honeycomb infill structure and systematically evaluated across multiple infill densities. The honeycomb structure offers considerable research potential due to its high specific strength (i.e., strength-to-weight ratio) and stiffness relative to conventional infill patterns, including grid, triangular, quadrangular lattice, and other energy-absorbing structures [69,70,71,72,73]. These properties make it particularly attractive for various biomedical applications.

The primary objective of the FEA simulations was to assess the influence of specimen geometry and infill density on fatigue crack growth resistance. The analysis was based on previously verified titanium alloy models [66,67,68], following the ASTM F382 standard [74]. These models were adopted as a foundation because they incorporate properly defined boundary conditions and loading configurations for four-point bending simulations.

## 2. Materials and Methods

The material selected for this study is the PLA polymer previously utilised in several research works [27,34,36,43,75,76,77], commercially designated as the “silk-grey” material produced by the German RepRap company (InnovatiQ GmbH, Kapellenstraße, Feldkirchen, Bavaria, Germany). The material has a density of 1.25 ± 0.05 g/cm^3^, and its tensile [27], impact [75,76], fracture [34,36], and fatigue properties [43] have already been investigated. In all these studies, the primary research focus was the influence of infill geometry on the resulting mechanical behaviour. The material model used in this research is detailed in [27], with key FDM parameters employed for model calibration summarised in Table 1.

**Figure 1:**
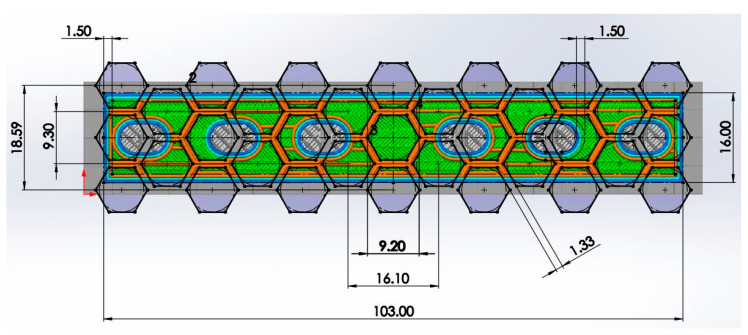
Slicer software image imported to the CAD software to reconstruct the infill architecture (the 20% infill case, units in mm).

**Figure 2:**
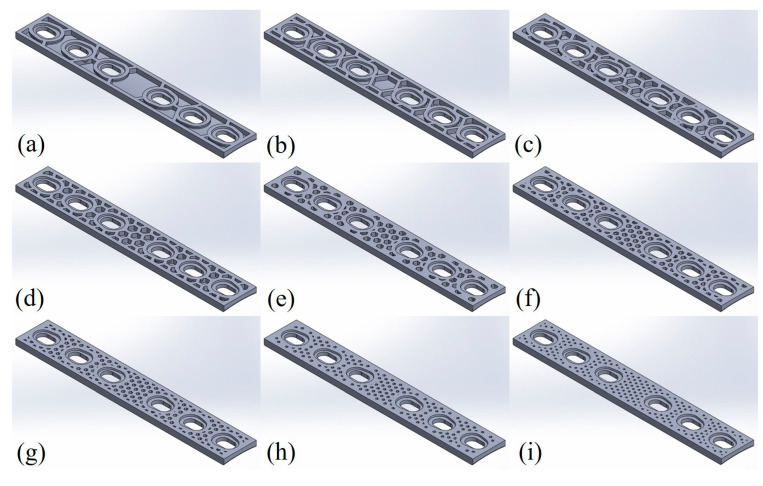
Orthopaedic plate layouts with considered infill architectures for: (**a**) 10%; (**b**) 20%; (**c**) 30%; (**d**) 40%; (**e**) 50%; (**f**) 60%; (**g**) 70%; (**h**) 80%; (**i**) 90% infill density.

To incorporate the infill structure into a previously studied titanium alloy orthopaedic plate design, the CAD model was prepared in SolidWorks^®^ 2022 (Dassault Systèmes, Vélizy-Villacoublay, France) based on the engineering drawing provided in Figure 12d of [67]. The bulk plate geometry was then imported into the Simplify3D slicer software (Simplify3D, Reed Hartman Hwy, Cincinnati, OH, USA) to generate the infill structure. A honeycomb infill pattern was applied, and the resulting slicer images were subsequently re-imported into SolidWorks^®^ to accurately reconstruct the infill architecture as it would be manufactured in PLA orthopaedic plates (see Figure 1). The reconstructed pattern was merged with the original CAD file, finalising the AM orthopaedic plate designs. Using this approach, ten geometries were created with infill densities ranging from 10 to 100% in increments of 10%. For all configurations, the envelope wall thickness was maintained at 1.5 mm (corresponding to two perimeter lines), while the honeycomb wall thickness was kept constant at 1.33 mm. The resulting layouts in SolidWorks^®^ for all considered infill cases are shown in Figure 2.

The models were considered idealised, neglecting potential manufacturing imperfections such as layer adhesion defects, internal voids, and raster path deviations, as their inclusions would significantly increase computational cost and complicate the analysis. Although FDM technology cannot achieve true 100% infill density in most cases, the bulk model was assumed to be fully dense for FEA purposes.

The fracture mechanical fatigue is described using Paris’ law (see [47]) with the corresponding C and m constants for PLA material listed in Table 2. These values differ slightly from those reported in [43], as additional specimens were tested to reinforce the findings. While Paris’ law assumes a linear–elastic fracture mechanism, which is reasonable for brittle PLA, it does not account for local plasticity, anisotropy, or printing defects inherent to the FDM process. These effects are acknowledged but intentionally excluded to maintain a consistent modelling framework for comparative evaluation of internal architectures.

The FEA simulations additionally required PLA material properties, which were experimentally determined in [27]. These properties are strongly influenced by printing parameters such as infill density, raster orientation, and layer height, as well as by process-induced structural imperfections inherent to FDM, including air gaps, interlayer discontinuities, and other defects [20,22]. For a full-volume specimen manufactured with a layer height of 0.1 mm, the measured ultimate tensile strength was 46.24 MPa, and the elastic modulus was 3.18 GPa. These experimentally obtained values were adopted as input parameters for the simulations. Although slightly lower than typical values reported for injection-moulded PLA, they are consistent with the mechanical properties of FDM-fabricated PLA, reflecting the combined influence of printing parameters and defect-related features, characteristic of AM materials [1,3,20,22,30].

## 3. Development of Numerical Models

**Figure 3:**
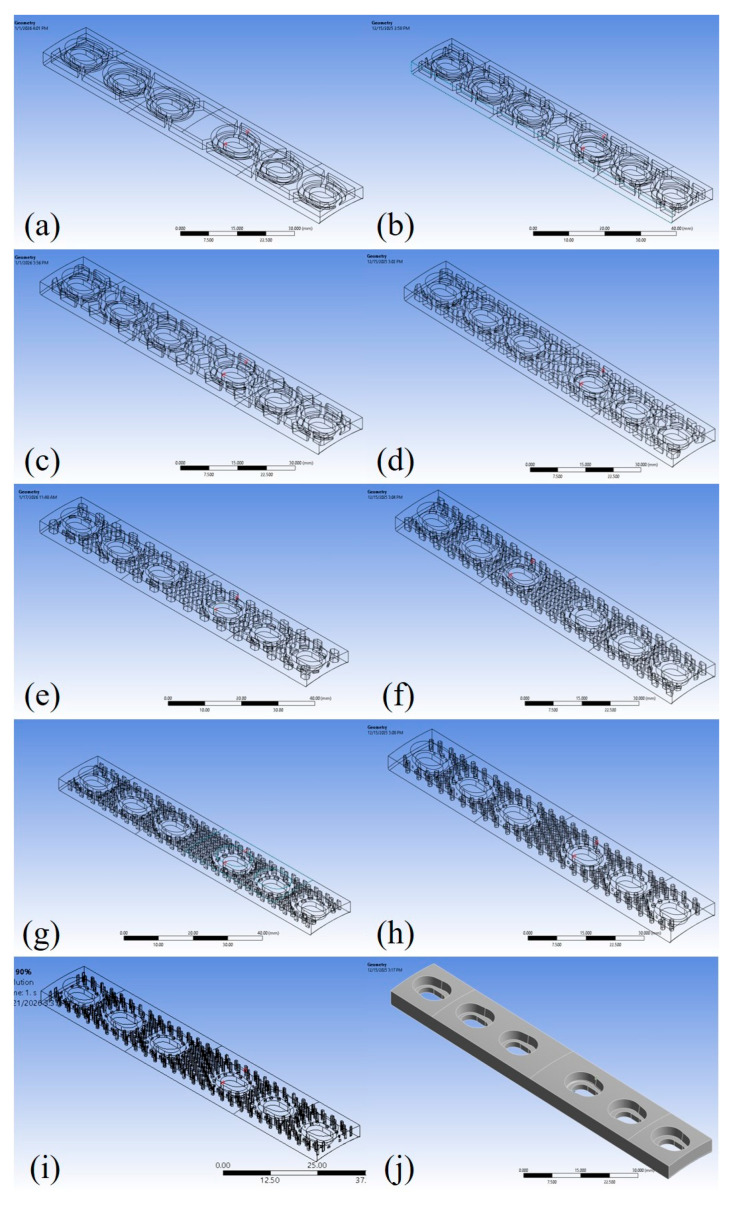
Orthopaedic plate numerical models in ANSYS for: (**a**) 10%; (**b**) 20%; (**c**) 30%; (**d**) 40%; (**e**) 50%; (**f**) 60%; (**g**) 70%; (**h**) 80%; (**i**) 90%; (**j**) 100% infill density.

The FEA simulations of fatigue crack growth in orthopaedic plates were performed using ANSYS 2022 R2 software (Ansys, Inc., Canonsburg, PA, USA) with the SMART crack option employed [78,79]. All models were based on the previously developed and validated configurations described in [66,68], with minor modifications to the geometry, material properties, and mesh size, to ensure proper convergence of the results. A total of ten models were considered, each with a different infill percentage ranging from 10% up to 100% (solid). Each model geometry is presented in Figure 3, highlighting the internal honeycomb-like structure for all configurations except for the 100% one. The SMART crack method assumes a linear–elastic, homogeneous material representation, although local crack propagation in FDM-grade PLA may be influenced by anisotropy and microstructural defects. The method focuses on assessing the relative fatigue behaviour across different infill configurations, providing a consistent comparative framework.

**Figure 4:**
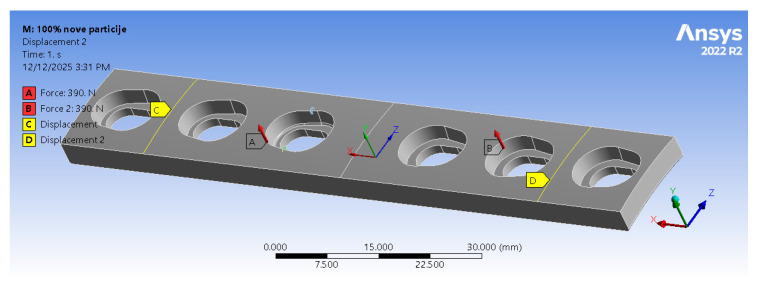
Applied loads (two forces) and boundary conditions in the FEA model.

Each model was simulated for a four-point bending scenario, with loads and boundary conditions defined accordingly [74]. Two linear segments of the top surface, where fatigue cracks were located, were constrained in the *y* and *z* directions, while translation along the *x* (longitudinal) axis was left free (see Figure 4). These constraints were applied only to stabilise the model and replicate the support conditions of the setup reported in [66,67,68], while minimising artificial stiffness effects. The constrained regions were positioned sufficiently away from the crack front to avoid influencing the local stress field and SIFs. To prevent loss of contact and excessive deformation, a remote displacement was introduced to the central section of the model, ensuring that deformation occurred only along the vertical (*z*) axis, corresponding with a physical four-point bending experiment. The load was applied as two forces acting on the opposite side of the model relative to the crack (Figure 4).

Although the load directions and locations were defined identically to those in [66,67,68], their magnitudes had to be substantially reduced because PLA is a significantly weaker material than titanium alloys. Applying the original experimental loads (3.9 kN) would have prevented successful fatigue crack simulations. Since the primary objective was to investigate the influence of internal architecture on the fatigue performance of orthopaedic plates, the load level was adjusted to provide relevant stress intensity factor (SIF) values and fatigue cycles. After several iterations, a 390 N load (10% of the original magnitude) was adopted. Since this study represents a comparative analysis rather than a direct prediction of fatigue life under real conditions, the load reduction was applied to ensure a meaningful and consistent comparison between selected internal architectures.

The finite element mesh was generated using the patch conforming method, with a default element size of 0.5 mm. Such a refined mesh was required to ensure solution accuracy, given the models’ geometric complexity. Mesh convergence was assessed by initially meshing the models with a default element size of 0.75 mm, which was then progressively reduced. An example of this procedure is presented in Table 3 for the 80% infill case. As the element size decreased, the predicted number of cycles and crack extension increased gradually, up to an element size of 0.6 mm. In the subsequent iteration, a further reduction in element size led to a decrease in both values, while the model with a 0.5 mm element size yielded results within the range defined by the 0.6 mm and 0.55 mm meshes. Based on these observations, an element size of 0.5 mm was considered sufficient to achieve mesh convergence and numerical accuracy.

**Figure 5:**
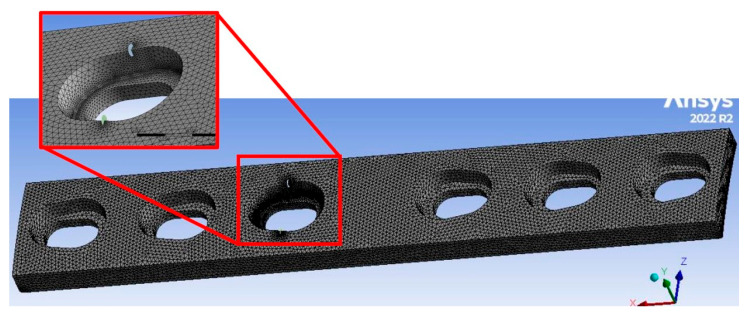
Mesh appearance for all models, with a magnified view of the opening containing circular cracks.

The same meshing strategy was applied to all models, except for the 100% infill configuration. Owing to the absence of a distinct honeycomb structure in this model, a slightly coarser mesh with an element size in the range of 0.6–0.7 mm was found to be sufficient. In accordance with the requirements of SMART crack growth simulations, all meshes consisted exclusively of tetrahedral elements. The models were discretised using the SOLID187 high-order three-dimensional 10-node finite element. Owing to its quadratic interpolation capabilities, this element type provides improved accuracy for curved geometries and is particularly suitable for irregular meshes generated from complex CAD models. An example of the generated mesh, highlighting the region around the initial cracks, is shown in Figure 5. Two initial cracks were positioned in the most critical area, specifically in the opening located near the midsection of the plate. Due to the model’s symmetry, only one of the fatigue cracks was monitored during the simulations. The initial cracks were idealised as circular shapes with a radius of 0.5 mm to provide a consistent starting point for the analysis, despite the real cracks in FDM-grade PLA components being typically irregular and strongly influenced by internal architecture and defects. To improve the accuracy of the results and better approximate the real scenario, fillets were applied to the edges of the opening. No further modifications were required for the other openings, as these regions were outside the scope of the presented simulations.

The number of load steps was determined iteratively for each model, ranging from 14 to 22, to obtain the fatigue crack growth up to the onset of the unstable crack propagation, corresponding to the impending failure of the plate. Each step was subdivided into multiple substeps, with a minimum of 5 and a maximum of 10, to ensure proper convergence during the calculations.

Using the default settings for load step timing in ANSYS often led to premature termination of the calculations; therefore, smaller load step intervals achieved by dividing them into substeps were necessary to maintain numerical stability.

## 4. Fatigue Crack Growth Simulation Results

**Figure 6:**
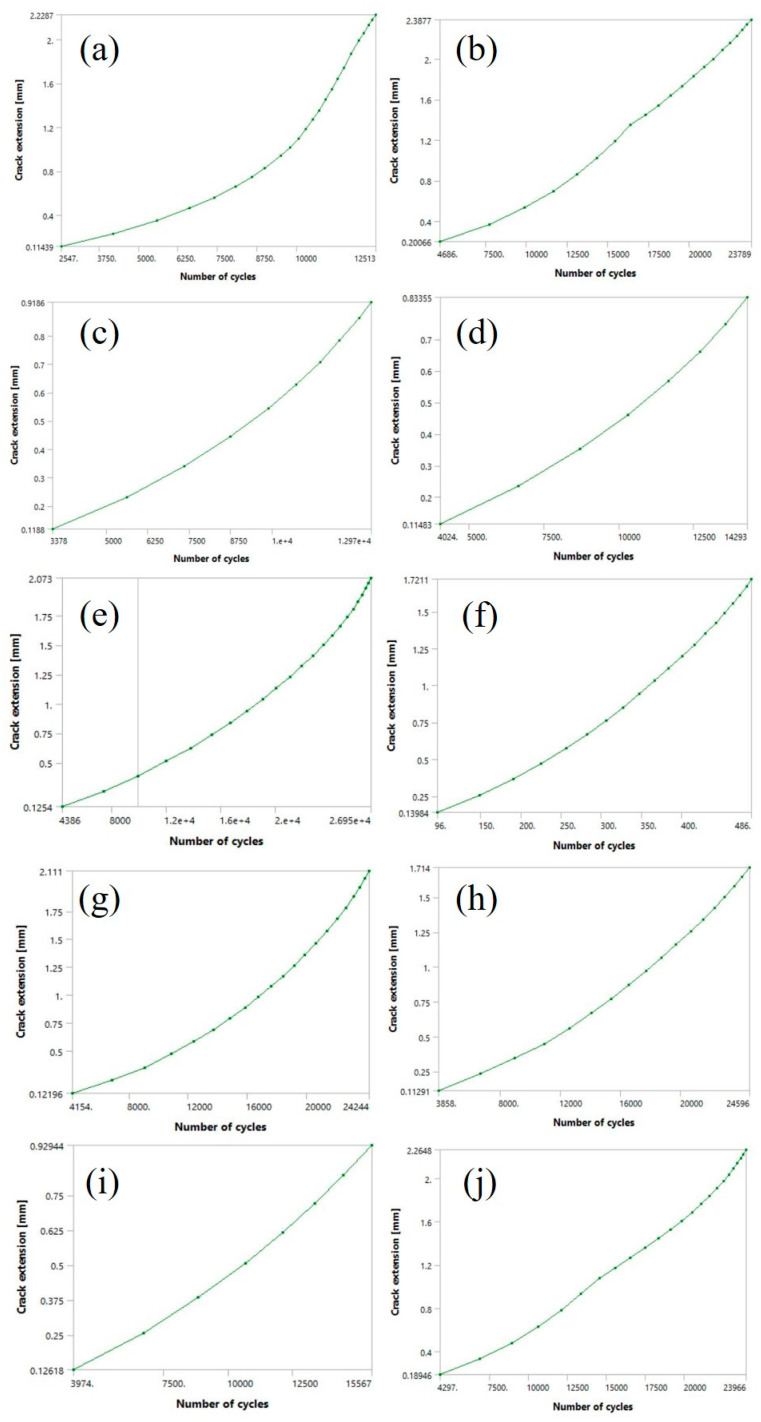
Number of cycles-crack extension (a-N) diagrams for: (**a**) 10%; (**b**) 20%; (**c**) 30%; (**d**) 40%; (**e**) 50%; (**f**) 60%; (**g**) 70%; (**h**) 80%; (**i**) 90%; (**j**) 100% orthopaedic plate models.

The results of SMART crack growth simulations are shown for all ten models, with the main focus on SIFs, crack growth, fatigue life, and their mutual relations. As anticipated, variations in model geometry had a significant impact on the outcomes, often different from conventional expectations for orthopaedic plate fatigue simulations. Figure 6 illustrates the relationship between total fatigue cycles and crack extension for each model.

As shown in Figure 6, the number of fatigue cycles did not decrease consistently with decreasing infill density, contrary to the expectation that reduced load-bearing cross-sections would result in shorter fatigue life. The differences between the 100% model and the 70% and 80% models were negligible in terms of the number of cycles, but more pronounced with respect to crack extension. The highest number of cycles was recorded for the 50% model, which endured 26,950 cycles before failure. Interestingly, the 20% model had a similar a-N diagram to the 100% model, despite having significantly less material. The 10%, 30%, and 40% models exhibited comparatively low remaining fatigue lives, with the number of cycles ranging between 12,000 and 14,500, while the 90% model showed a substantially lower cycle count compared to the 100% model. The lowest fatigue performance was unexpectedly delivered by the 60% model, with a remaining fatigue life two orders of magnitude lower than the other models (i.e., 486 cycles), while its crack extension was comparable to that of the 80% model (~1.7 mm).

The considerable crack extension was observed in the 10%, 20%, 50%, 70%, and 100% models, with values exceeding 2 mm. Among these, the highest crack extension was recorded for the 20% model, reaching 2.39 mm. In contrast, the lowest crack extension was observed in the 30%, 40%, and 90% models, none of which reached 1 mm. The minimum value was obtained for the 40% model (0.83 mm).

Regarding SIFs, the 50% and 100% infill models exhibited relatively similar values of 267.11 MPa·√mm and 228.49 MPa·√mm, respectively. Noticeably lower SIFs were measured in the 30%, 40%, 80% and 90% models, consistent with their relatively short fatigue crack lengths. The 90% model has the lowest SIF values overall (~109 MPa·√mm). The 10%, 20%, 50%, 70%, and 100% models form a separate group, with SIFs ranging from 170 to 300 MPa·√mm and crack extensions exceeding 2 mm. The 60% model exhibited unusual behaviour, with SIFs reaching values as high as 681.29 MPa·√mm—nearly three times higher than the 100% model and approximately six times higher than the 90% model. This substantial increase in SIFs during the fatigue crack growth simulation accelerated crack propagation, resulting in a markedly lower number of cycles compared to the other models. This anomalously high SIF value is attributed to local geometric effects, specifically the proximity of the honeycomb internal structure to the propagating crack, rather than to the intrinsic material properties of the PLA material.

**Figure 7:**
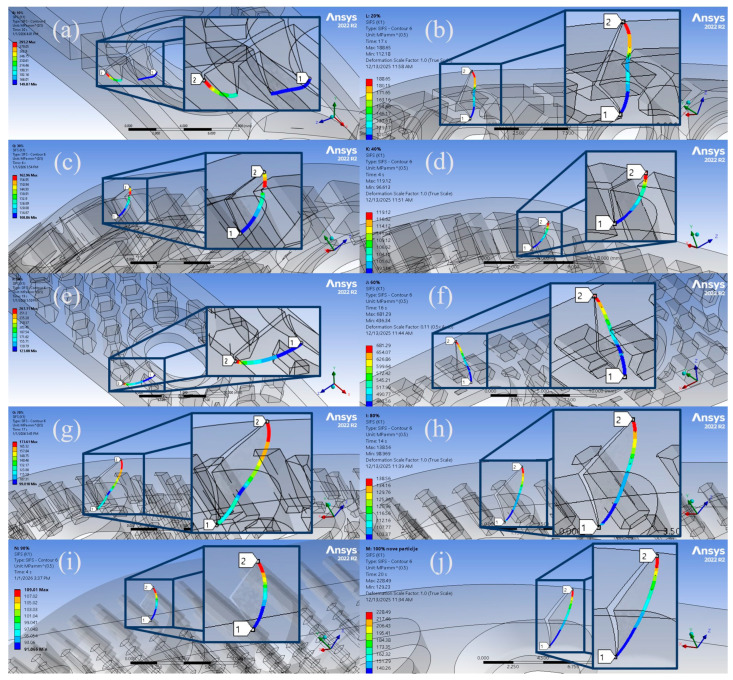
SIFs along the crack front for: (**a**) 10%; (**b**) 20%; (**c**) 30%; (**d**) 40%; (**e**) 50%; (**f**) 60%; (**g**) 70%; (**h**) 80%; (**i**) 90%; (**j**) 100% orthopaedic plate models.

Table 4 summarises all the aforementioned results, while the SIFs along the crack front for all considered numerical models are presented in Figure 7. All images in the figure are magnified to provide a clearer view of the SIF distribution. As shown, high SIF values are observed on the top surface of the orthopaedic plate, resulting from the gradual transition of the crack front geometry from a circular to an elliptical shape.

**Figure 8:**
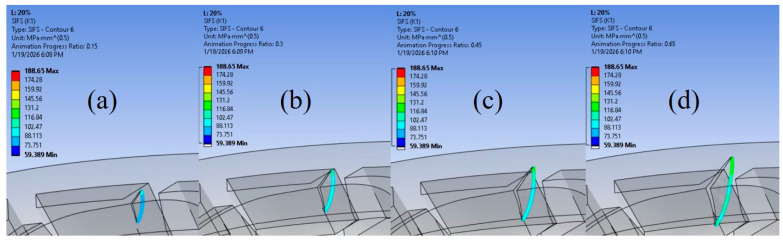
SIF values and their distribution for the 20% infill orthopaedic plate model at crack extensions of: (**a**) 0.5 mm; (**b**) 1 mm; (**c**) 1.5 mm; (**d**) 2 mm.

The numerical simulations also enabled the identification of the moment when the fatigue crack transitioned from a circular to an elliptical shape, corresponding to the onset of unstable crack growth. An example of this is shown in Figure 8 for the 20% infill density model, which was selected due to its pronounced change in crack geometry. The figure is divided into four stages, ranging from 0.5 mm to 2 mm of crack growth. In this case, crack propagation along the horizontal (*z*) axis reached the end of the plate, similar to the 100% model, and clearly dominated over vertical (*y*) axis propagation. The transformation of the crack front shape is particularly evident in the 2 mm crack growth image, marking the initiation of the unstable fatigue crack propagation stage.

## 5. Discussion

By comparing the obtained results of the fatigue crack growth simulations, it is evident that the internal architecture of the models had a far more pronounced effect on crack propagation than the infill density. The location and geometry of internal holes within the cross-section, particularly relative to the crack initiation site, played a critical role. The internal holes in this case originate from the geometric features of the hexagons that make up the honeycomb structure.

For the solid 100% infill model, the fatigue crack propagated without obstruction, reaching a maximum crack extension of 2.26 mm after 23,966 cycles. Similar behaviour was observed in the 20% and 80% infill models, where cracks were located farther from internal holes, allowing unobstructed crack propagation through the material. This resulted in increased crack extension and higher fatigue life, with the only notable difference being the slightly lower crack extension in the 80% model (~1.7 mm) compared to approximately 2.3 mm for the 20% and 100% models. In all three cases, the cracks evolved into a distinctly elliptical shape, driven by unimpeded horizontal crack propagation.

The 70% infill model showed similar properties, with a crack extension of over 2 mm and a remaining fatigue life comparable to the 80% and 100% models, further confirming that minor reductions in material content do not necessarily compromise fatigue performance if internal holes do not interfere with the crack path.

**Figure 9:**
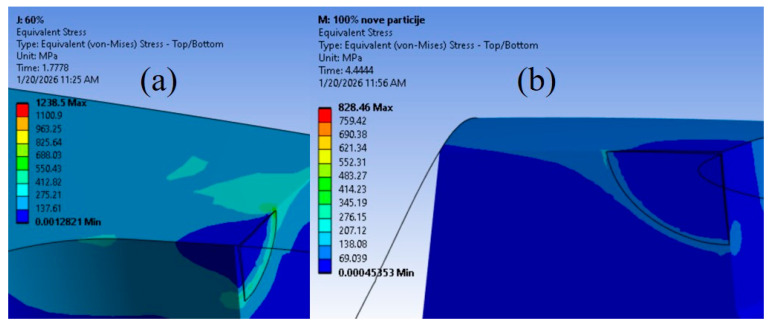
Comparison of stress distribution in: (**a**) 60%; (**b**) 100% infill model case.

In contrast, the 60% infill model presented an anomaly. Although its crack extension is comparable to the other models, the number of fatigue cycles is drastically reduced (486), nearly 50 times lower than the maximum observed values. With respect to geometric influence, it should be noted that in this case, the fatigue crack did not intersect any of the internal holes; therefore, direct interaction with internal structure cannot explain the unexpectedly low number of fatigue cycles. However, the initial crack was located in proximity to the holes, indicating that crack propagation occurred in regions with elevated stress concentration induced by geometric discontinuities. This promoted accelerated crack growth and accounts for the extreme SIF values observed along this crack front. An observation of the stress distribution in the vicinity of the crack front, compared with the other models, confirms that stresses in the 60% model were indeed substantially higher. This can be seen in Figure 9, which presents sectional views of the 60% and the 100% plate models. The stresses around the crack tip in 60% model exceeded 500 MPa at the very onset of fatigue crack growth, whereas the corresponding stresses in the 100% model were approximately 160 MPa. These results indicate that the proximity of an internal hole within the cross-section, and the associated stress concentration it induces, plays a more critical role in determining the remaining fatigue life than the mere presence of a hole along the crack propagation path.

**Figure 10:**
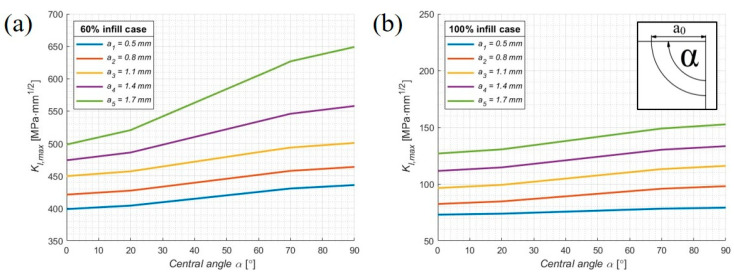
SIF values along the crack front as a function of the central crack angle for the orthopaedic plate models with: (**a**) 60%; (**b**) 100% infill.

**Figure 11:**
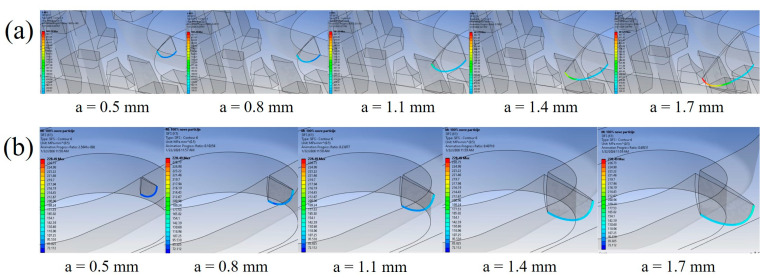
Crack front evolution at selected crack extensions for: (**a**) 60%; (**b**) 100% infill orthopaedic plate models.

The analysis can be further strengthened by examining the SIF distribution along the crack front with respect to the central crack angle, following the approach of [80]. Five crack fronts were analysed, corresponding to crack extensions ranging from 0.5 mm to 1.7 mm. As shown in Figure 10, the SIF values at the onset of crack propagation in the 60% model are substantially higher than the maximum SIF values observed in the 100% infill model. Furthermore, the charts reveal a sudden increase in SIF values for the 60% model after approximately 1.5 mm of crack propagation at the top surface of the plate. The initial crack configuration, including the definition and orientation of the central angle α is illustrated in the top-right corner of Figure 10b. This behaviour indicates a transition of the crack front from a cylindrical to an elliptical shape, marking the onset of unstable crack growth and leading to a drastic reduction in the remaining fatigue life of the component. In contrast, the 100% infill model exhibits a more gradual and stable evolution of SIF values along the crack front. The corresponding ANSYS visualisations for the five considered crack extensions are presented in Figure 11.

The 50% infill model provided an interesting outcome, achieving the highest number of cycles among all ten models (26,950) with a crack extension of 2.07 mm, outperforming the 80% and 100% infill models, in which the internal geometry had little to no influence on crack propagation. This result demonstrates that careful optimisation of the internal architecture can significantly enhance fatigue performance, even with a reduced material volume. A lack of strict correlation between infill density and the observed results has also been reported in the literature [76]. Furthermore, the higher SIF values observed in the 50% infill model, compared to the 100% infill case, can be attributed to the fact that the fatigue crack not only reached the end of the plate but also began propagating in the perpendicular direction—a behaviour not observed in the 100% infill model.

For the 40% infill model, crack propagation was impeded by a nearby honeycomb hole, causing horizontal growth to halt at that location. Consequently, the crack retained a circular shape, unlike in the other models. For the same reason, the number of cycles was lower (~14,300), as crack propagation terminated earlier, roughly 40–45% fewer than in the best-performing models. This also accounts for the more uniform SIF distribution observed along the crack front, as this was the only configuration in which the fatigue crack maintained its initial circular geometry. In contrast, the other models developed distinctly elliptical crack fronts, resulting in higher SIFs, particularly in cases where horizontal crack propagation reached the end of the plate, as observed in the 20% and 100% infill models.

Lower infill models further highlight the critical role of hole location. In the 30% infill model, the crack propagated toward an internal hole but was arrested, resulting in a total extension of 0.92 mm and ~13,000 cycles. The 10% infill model, despite having the lowest material content, allowed the crack to propagate fully through the cross-section, reaching an extension of 2.23 mm and roughly 12,500 cycles. These results emphasise that fatigue performance depends not only on the amount of material in the component but also on the interaction between the crack path and internal holes.

Finally, the 90% infill model exhibited behaviour similar to that of the 30% case. The crack propagation was interrupted by an internal hole, halting at approximately 0.9 mm and ~15,500 cycles. The influence of hole geometry and distribution is particularly evident when considering that the model with the lowest material content (10% infill) achieved a remaining fatigue life comparable to that of the model with the second-highest infill (90%).

Overall, these results demonstrate that fatigue behaviour is strongly influenced by internal geometry and hole distribution, often more so than by infill density. Holes located near crack initiation sites can dramatically reduce fatigue life, as in 60% infill case, while models with strategically positioned holes can maintain or even improve performance despite reduced material content.

## 6. Conclusions

The research presented in this paper involved numerical analyses of the fatigue behaviour of orthopaedic plates manufactured from PLA polymer with varying infill levels, to evaluate the influence of infill density on fatigue crack growth resistance. All numerical models were derived from previously developed and validated FEA plate models [66,67,68], which served as a reliable foundation for the present study. However, due to the substantially lower mechanical properties of PLA compared to metallic materials, the standard load magnitudes commonly used in such analyses could not be applied and had to be significantly reduced. Consequently, the results obtained for the PLA models should be interpreted solely in terms of the influence of geometry on fatigue crack growth and should not be considered as confirmation for actual load-bearing orthopaedic applications.

Based on the results obtained for fatigue crack growth, fatigue life, and SIFs across ten different infill density cases, the following conclusions can be drawn:

- The infill density itself has a less pronounced effect on fatigue performance than the spatial distribution of internal holes relative to the initial crack location. For example, models with 100% and 80% infill had comparable fatigue performance to the 20% model, due to the crack path being unobstructed by any of the internal holes.
- Crack propagation occurring in the vicinity of the internal holes, as observed in the 60% infill case, resulted in significantly inferior fatigue performance compared to cases where the crack propagated toward the internal hole. This resulted in a fatigue life that was 30–50 times shorter than that of the remaining configurations. This can be attributed to substantially increased stress concentrations near the initial crack caused by the proximity of internal holes in the cross-section.
- The 50% infill model achieved the highest fatigue life among all ten configurations, demonstrating that optimisation of the internal structure can substantially enhance fatigue performance, even with less material. The 20%, 70%, and 80% infill models also showed good performance in terms of fatigue life and crack extensions.
- The 90% infill model exhibited unexpectedly poor performance, as crack propagation was interrupted by an internal hole, resulting in fatigue life comparable to the lowest-performing 10%, 30%, and 40% infill models.
- In most cases, the fatigue crack propagated to the end of the plate. Monitoring the evolution of the crack shape allowed clear identification of the transition from an initial circular to an elliptical shape, marking the onset of unstable crack growth.

The proposed methodology can be extended to other material types, as its applicability has been demonstrated for two fundamentally different classes of materials: a metallic alloy [66,67,68] and a polymer. This study highlights the critical role of internal structure in determining the fatigue performance of an orthopaedic plate design. The present research serves as a foundation for the development of high-strength PLA-based materials and composites as potential alternatives to conventional materials, such as titanium alloys, in biomedical engineering applications, including orthopaedic plates [81,82].

A key focus for future research will be the shape and distribution of internal holes, as optimisation of the internal geometry has the potential to further enhance fatigue life, as evidenced by the superior performance of the 50% infill model in this case study. Advanced approaches, such as graph neural networks (GNNs) or other infill structure generation approaches [83,84,85], may be employed to achieve optimal internal designs. Furthermore, extensive experimental validation of the proposed plates will be conducted in future studies.

## Figures and Tables

**Table 1:** Manufacturing parameters considered for PLA in this study.

Manufacturing Parameter	Value and Description
Layer height [mm]	0.1
Nozzle diameter [mm]	0.4
Nozzle temperature [°C]	200
Bed temperature [°C]	60
Speed [mm/s]	40
Raster orientation	Rectilinear
Infill pattern	Honeycomb
Infill density range [%]	10–100

**Table 2:** PLA Paris’ law constants.

Constants	Value
C [-]	1.8498 × 10^−4^
m [-]	3.2904

**Table 3:** Result convergence for the 80% infill model.

Finite Element Size [mm]	Number of Cycles [-]	Crack Extension [mm]
0.75	23,881	1.666
0.70	24,143	1.737
0.65	24,789	1.741
0.60	24,926	1.778
0.55	24,392	1.711
**0.50**	**24,596**	**1.714**

**Table 4:** Overview of results of fatigue crack growth simulations for all infill models (10–100%).

Plate Model (Infill Percentage)	Number of Cycles [-]	Crack Extension [mm]	Stress Intensity Factor [MPa·√mm]
10%	12,513	2.23	295.2
20%	23,789	2.39	188.65
30%	12,970	0.92	162.96
40%	14,293	0.83	119.12
50%	26,950	2.07	267.11
60%	486	1.72	681.29
70%	24,244	2.11	173.61
80%	24,596	1.71	138.56
90%	15,567	0.93	109.01
100%	23,966	2.26	228.49

## Data Availability

The original contributions presented in this study are included in the article. Further inquiries can be directed to the corresponding authors.
